# Clinical efficacy on acupuncture for perennial allergic rhinitis: a study protocol for a randomized clinical trial

**DOI:** 10.3389/falgy.2025.1600032

**Published:** 2025-06-16

**Authors:** Jia-xin Yang, Shu-ren Ming, Hui Chen, Yue-lai Chen

**Affiliations:** ^1^ Longhua Hospital, Shanghai University of Traditional Chinese Medicine, Shanghai, China; ^2^ Yueyang Hospital of Integrated Traditional Chinese and Western Medicine, Shanghai University of Traditional Chinese Medicine, Shanghai, China; ^3^ Shanghai Literature Institute of Traditional Chinese Medicine, Shanghai, China

**Keywords:** acupuncture, allergic rhinitis, sham acupuncture, efficacy, life quality

## Abstract

**Background:**

Allergic rhinitis (AR) is a prevalent allergic disorder. Acupuncture has been widely utilized to alleviate allergic symptoms, and numerous studies have investigated its therapeutic effects on AR. However, due to the challenges associated with establishing appropriate placebo controls, few studies have directly compared acupuncture with sham acupuncture for AR treatment. This trial investigates the comparative effectiveness and tolerability of acupuncture vs. placebo needling for allergic rhinitis patients.

**Methods:**

This clinical trial features a stratified randomization scheme with 1:1 allocation, single-blind assessment, and a total sample size of 84 participants. After screening for inclusion, qualified subjects with perennial allergic rhinitis will be randomly allocated to treatment group(accepting acupuncture, *n* = 42) or control group (accepting sham acupuncture, *n* = 42). The intervention will last over a 4-week period. The main efficacy outcome is the change in main symptom severity assessed by the Visual Analogue Scale (VAS) after each week of treatment. Secondary outcomes include the Total Nasal Symptom Score (TNSS), Efficacy Index (%) after each treatment session, time to onset of effect, Rhinitis Quality of Life Questionnaire (RQLQ) scores after each week of treatment, and the additional use rate of anti-allergic medications.

**Conclusion:**

The findings of this study aims to evaluate the effectiveness and safety of acupuncture in treating perennial allergic rhinitis through comprehensive assessment of symptom relief, time-effect relationships, quality of life improvements, and reduction in anti-allergic medication use.

**Trial Registration:**

Chinese Clinical Trial Registry (ChiCTR2400086227).

## 1 Introduction

Allergic rhinitis (AR) is a non-infectious inflammatory disease characterized by symptoms such as sneezing, rhinorrhea, nasal congestion, and nasal pruritus. The prevalence of self-reported AR ranges from approximately 2%–25% in children and 1%–40% in adults (1, 2). Based on the timing and type of allergen exposure, AR is classified into seasonal allergic rhinitis (SAR), typically triggered by outdoor allergens such as pollens or molds, and perennial allergic rhinitis (PAR), commonly caused by indoor allergens like house dust mites, molds, cockroaches, and animal dander (3, 4). Although non-life-threatening, AR significantly impairs quality of life, work/school performance, and in children, facial and vocal development (5, 6). In addition, AR patients frequently experience mental health issues, including insomnia, anxiety, depression,stress and suicidal, impair cognitive function, and contribute to fatigue, irritability, and reduced quality of life (7). AR also association with comorbidities like asthma underscores the need for effective treatments (8). Current pharmacotherapy (antihistamines, corticosteroids, leukotriene antagonists, etc.) provides rapid but transient symptom relief, often with side effects (9–11). Symptoms typically recur after discontinuation. While immunotherapy can modify disease progression, its months-to-years duration challenges patient compliance. Most patients use medications only intermittently for severe symptoms, highlighting the urgent need for safer, more sustainable therapies to improve long-term outcomes.Acupuncture,as an important therapy in traditional Chinese medicine, has been used for centuries to treat nasal disorders. In 2015, the American Academy of Otolaryngology-Head and Neck Surgery recommended acupuncture as an alternative treatment for AR (12). Our previous study (13) demonstrated that a 10-day course of acupuncture significantly reduced the severity of major nasal and ocular symptoms (*P* < 0.05). Building on these findings, we designed a single-blinded, randomized, controlled trial with an extended treatment duration to assess the effectiveness and safety of acupuncture in managing AR.

## 2 Methods and analysis

### 2.1 Study design and setting

**Figure 1:**
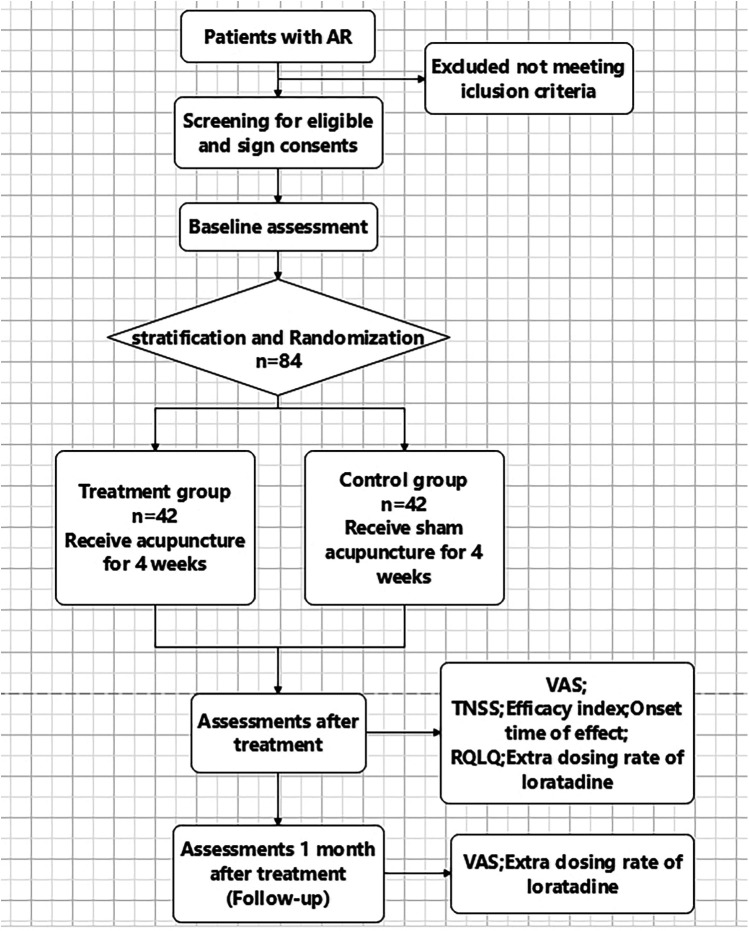
Flow chart.

This is a stratified, randomized, single-blinded, controlled trial. The study flowchart is presented in Figure 1, and the schedule of enrolment, interventions, and assessments is presented in Table 1. The study sets in acupuncture department of Yueyang Hospital of Integrated Traditional Chinese and Western Medicine, Shanghai University of Traditional Chinese Medicine, China.

**Table 1:** Schedule of enrolment, interventions, and assessment.

Study periosd	Erolment (0 week)	Intervention period (1–4 weeks)		Follow-up period
	Before treatment	After everytime treatment	After weekly treatment	4 weeks after the end of treatment
Eligibility screening	×			
Sign informed consent	×			
Baseline information collection	×			
Randomization	×			
Intervention				
Acupuncture		×		
Sham acupuncture		×		
Outcomes				
Symptoms VAS	×		×	×
TNSS	×	×		
Efficacy index		×		
Onset time of effect		×		
RQLQ	×		×	
Extra dosing rate of loratadine		×		×
Safety evaluation		×		

### 2.2 Participant and consumer involvement

Inclusion criteria includes a diagnosis of perennial allergic rhinitis (14), between the age of 18 and 60. All participants will sign consent documents before participation. Exclusion criteria includes: Severe comorbidities (e.g.,uncontrolled hypertension, diabetes, malignancies, organ dysfunction).Nasal abnormalities, sinusitis, asthma, or prior nasal surgery. Recent use of corticosteroids/Antihistamines (1 month) or immunotherapy (1 year). Prior acupuncture experience or pregnancy/lactation. Withdrawal Criteria includes using rescue medicine 3 times/week,rescue medicine-related Adverse Event ≥Grade 2 (CTCAE v5.0); experiencing severe complications or other serious illnesses and is unable to continue cooperation. Data were analyzed by intention-to-treat (ITT).

All participants provided informed consent and could withdraw anytime. Data were used solely for research purposes.

### 2.3 Acupoint selection

**Figure 2:**
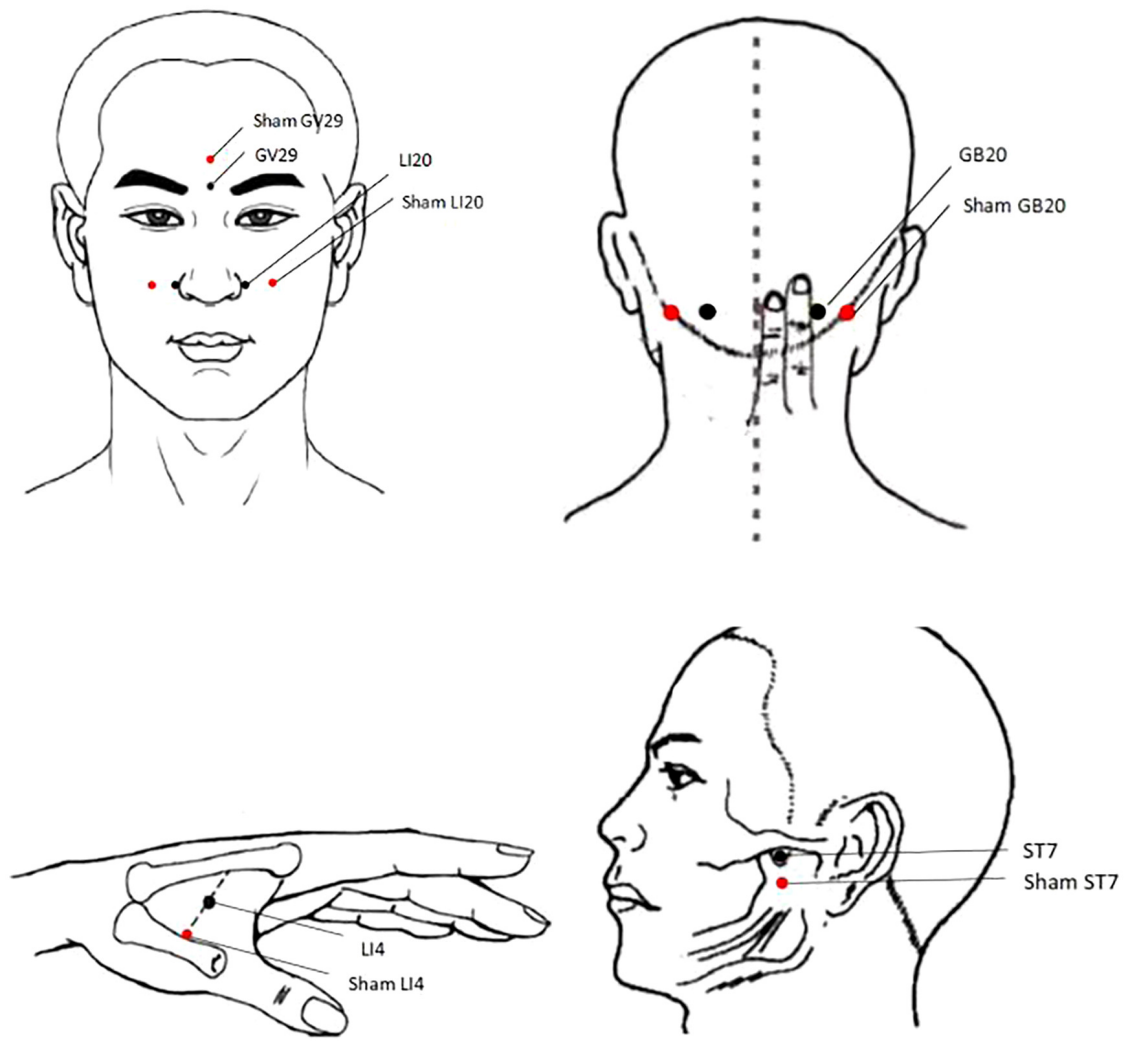
The location of acupoints and sham acupoints.

Acupoints: LI20, ST7, GB20, LI4, and GV29 on both side (Figure 2 and Table 2) were selected based on the following rationale: (1) Acupoints for allergic rhinitis recommended in textbooks “Acupuncture” (China Traditional Chinese Medicine Press, 11th Edition) (2) Effective acupoint combinations validated in our previous clinical studies. To ensure standardization and reproducibility of the protocol, we have adopted a fixed acupoint prescription without individualized modifications. While this approach may not fully align with the traditional Chinese medicine principle of individualized treatment based on syndrome differentiation, it enhances the internal validity of our research by allowing assessment of a specific acupoint combination.

**Table 2:** The locations of acupoints and sham acupoints.

Acupoint	Location	Sham acupoint	location
LI20	Location in the nasolabial groove,level with the midpoint of the lateral border of the ala nasi	Sham LI20	20 mm lateral to LI20
ST7	Acupuncture Point Location On the face, anterior to the ear, in a depression between the zygomatic arch and the mandibular notch, with mouth closed.	Sham ST7	20 mm below ST7
GB20	On the nape, below the occiput, at the level of DU 16, in the depression between the upper portion of the sternocleidomastoid muscle and the trapezius	Sham GB20	20 mm lateral to GB20
LI4	On the dorsum of the hand,between the 1st and 2nd metacarpal bones, approximately in the middle of the 2nd metacarpal bone on its radial side	Sham LI4	20 cm on the radial side of LI4
GV29	On the forehead, at the midpoint between the two medial ends of the eyebrow.	Sham GV29	20 mm straight up to GV29

### 2.4 Operator qualifications and standardization

All acupuncture procedures will be performed by three licensed acupuncturists with at least 5 years of clinical experience, each holding nationally recognized certification in acupuncture practice. Prior to study commencement, all operators will undergo a one-week standardization training program covering acupoint location, needle insertion depth, manipulation techniques, and safety protocols. Following training, operator consistency will be evaluated through simulated treatment sessions, requiring at least 85% concordance in key operational parameters (e.g., acupoint location accuracy, needle insertion depth, manipulation frequency) among operators.

### 2.5 Interventions

Both groups will undergo 12 sessions with eye masks. Participants in the treatment group will undergo acupuncture by 0.25 × 40 mm disposable needles (Guizhou Andy Pharmaceutical Equipment Co., Ltd., Guizhou, China). Following skin sterilization, aseptic adhesive pads will be positioned GB20 on both sides, and acupuncture needles are inserted through the pads to a depth of roughly 10–15 mm, following the direction of the nasal tip. Upon needle placement, gentle and uniform twirling at a frequency of approximately 60 rotations per minute, with a rotation angle of approximately 180°-360° (STRICTA 2010 guidelines) are performed to achieve *de qi* (a characteristic sensations encompassing soreness, numbness,fullness, and a heavy feeling), which is regarded as a crucial factor for therapeutic effectiveness in acupuncture. The needles are then retained at GB20. Subsequently, aseptic adhesive pads are placed on bilateral LI20, ST7, LI4, and GV29. Acupuncture needles are inserted through the pads to depths of 10–20 mm (10 mm for LI20 and GV29; 20 mm for ST7 and LI4), following the direction of the nasal tip. After insertion, twirling manipulations are performed on all needles to achieve *de qi*. All needles are retained for 20 minutes.

Participants in the control group will receive sham acupuncture using pragmatic placebo needles (0.25 mm in diameter × 30 mm; “Andy” brand disposable sterile flat-head needles; Guizhou Andy Pharmaceutical Equipment Co., Ltd.) at non-acupoint locations (Figure 2 and Table 2). The procedures and treatment settings in the control group are identical to those in the treatment group,with the exception that neither skin penetration nor needle manipulation to elicit *de qi* is administered.

Both procedures follow Liu's research (15) and SHARE guidelines (16).

### 2.6 Rescue medication

In case participants experience any of the following conditions:VAS score ≥7 for any of the primary nasal symptoms for >2 hours;≥10 sneezing episodes within a 24-hour period;Complete nasal obstruction affecting sleep;Symptoms subjectively evaluated by the patient as severely affecting daily activities or work and becoming intolerable.Supplemental loratadine (Xuesu Shanghai Haini Pharmaceutical Co., LTD, Shanghai, China) will be administered 10 mg every day. All investigators and participants must systematically document: (1) All instances of rescue medication including timing, dosage, and reason for use (2) Adverse reactions to rescue medication including symptom description, severity, duration, assessment of relatedness to the rescue medication, and management measures.

These measures ensure patient safety while maintaining treatment efficacy assessment integrity. Collected data will inform comprehensive therapeutic outcome analyses. Participants will be instructed to refrain from using rescue medication for 24 hours prior to Primary outcomes assessment; If a participant has used rescue medication within 24 hours before a scheduled assessment, the assessment will be postponed until at least 24 hours after the last dose. And rescue medication use will be incorporated as a covariate for both primary and secondary outcomes. This approach adjusts its potential confounding effects on symptom scores. To ensure robustness, we will conduct sensitivity analyses. Besides, we will also do exploratory subgroup analysis stratified by whever they use rescue medication or not. All results will be interpreted with this limitation explicitly stated in the Discussion.

### 2.7 Outcome measures

#### 2.7.1 Primary outcome

Participants will self-report symptom intensity scores before the first treatment and after weekly treatments during the trial. The participants are required to self assess the severity of their four nasal symptoms (sneezing, watery rhinorrhea, nasal congestion, and nasal itching) and 4 non-nasal symptoms (ocular itching, watering, redness, itching of the ears, and/or palate) by using a 10-cm Visual Analogue Scale (VAS) (17) that ranges from “no symptoms” to “worst symptoms ever”. Scores will be assessed at baseline (before treatment initiation) and after each week of treatment.

#### 2.7.2 Secondary outcome

1. The participants self assess the four nasal symptoms (nasal obstruction, rhinorrhea, sneezing, and itching) involved in assigning the total nasal symptom score (TNSS) (18). The symptoms were graded on a five-point scale (where 0 indicates no symptoms, a score of 1 for mild symptoms that are easily tolerated, 2 for awareness of symptoms which are bothersome but tolerable and 3 is reserved for severe symptoms that are hard to tolerate and interfere with daily activity). Score will be calculated before the treatment and after everytime treatment.
2. The efficacy was evaluated according to the improvement of TNSS score

Efficacy index (%) = (TNSS total score before treatment-total score after treatment)/ total score before treatment ×100% (19).

Obvious effect: the nasal symptoms were significantly improved after treatment, and the curative effect was >76%;

Effective: Nasal symptoms improved after treatment, 26%≤curative effect ≤76%;

Ineffective: Nasal symptoms did not significantly reduce after treatment, and the curative effect was <26%.

3. Onset time of effect

After the treatment begin, the number of treatments will be recorded when the Efficacy index reaches 26%.

4. Quality of life score of AR patients:

The participants self assess symtoms by using the Rhinitis Quality of Life Questionnaire (RQLQ) (20), which has 28 questions in 7 items ranked from 0 to 6.

Score will be calculated before the treatment and after weekly treatment.

5. Safety evaluation

Adverse events (AEs) will be recorded on the Case Report Form (CRF), including the initial symptom presentation date, recovery timeline, intensity grading, treatment-associated occurrences, and final outcome status (resolution or persistence). AEs associated with acupuncture include needle stagnation, needle fainting, hematoma, or other discomfort. In the event of AEs, the research team will administer necessary medical care and provide corresponding reimbursement, while simultaneously notifying the Data and Safety Monitoring Board for documentation and review.

### 2.8 Data analysis

#### 2.8.1 Sample size calculation

The sample size calculation was determined through *a priori* power analysis conducted with PASS (v15.0.5, NCSS, LLC). Using a two-tailed alpha level of 0.05% and 90% statistical power, with equal group allocation (1:1), the computation was derived from the change in the AR symptoms VAS scores after treatment. Preliminary data indicated mean VAS scores of 33.6 (SD = 5.2) and 29.4 (SD = 4.8) for the treatment and control group, respectively. Accounting for a 20% dropouot rate, each group required 33 participants, yielding a total target enrollment of 84 patients.

#### 2.8.2 Randomization

The randomization sequence was generated by an independent statistician using SAS version 9.4 (SAS Institute, Cary, NC, USA) with a block randomization algorithm (block sizes of 4 and 6) to ensure balanced allocation between groups. Participants were stratified by disease severity into two strata (mild vs. moderate-to-severe) according to the ARIA guidelines (2016 revision) (14), Within each stratum, participants were randomized 1:1 to either the treatment or control group. The randomization sequence was concealed in opaque, sequentially numbered envelopes, which were opened only after baseline data collection to ensure allocation concealment (21). To ensure baseline comparability between groups, we monitored the distribution of key variables, including age (18–40 vs. 41–60 years), sex, disease duration (≤5 vs. >5 years), and baseline VAS scores. Any significant imbalances detected during the study were adjusted for in the final analysis using appropriate statistical methods.

#### 2.8.3 Blinding

To maintain blinding integrity, all investigators will undergo standardized training and adhere to the institutional segregation protocol. All study personnel—including participants, outcome evaluators, and statisticians—remain unaware of group assignments,except the acupuncturist. After the whole treatment period, blinding efficacy will be assessed by asking participants: “What kind of treatment do you think you received? (a) Real acupuncture, (b) Sham acupuncture, (c) Unsure, (d) Not caring.” The success of blinding will then be evaluated using Bang's blinding index (22).

#### 2.8.4 Analytical methods

Statistical analyses will be conducted utilizing the SAS software package, with statistical significance defined as *P* < 0.05. For continuous data following normal distribution, results will be reported as mean ± SD; non-normally distributed data will be presented as median (IQR). Continuous variables will be assessed by the *t*-test or Mann–Whitney *U*-test. Categorical data will be summarized as frequencies (percentages) and evaluated through *χ*^2^ tests or Fisher's exact tests. Additionally, time-by-treatment interactions will be analyzed employing generalized estimating equations or mixed-effects models. All statistical tests will use a 95% confidence level, considering results significant when *P* values are below 0.05.Rescue medication use will be incorporated as a covariate. To ensure robustness, we will conduct sensitivity analyses. Besides, we wil also do exploratory subgroup analysis stratified by whever they use rescue medication or not.

## 3 Discussion

Allergic rhinitis (AR), the most prevalent allergic disorder globally, remains challenging to manage effectively despite its non-life-threatening nature compared to other medical conditions. This chronic condition significantly impairs patients’ overall health and quality of life, with symptoms extending beyond classical nasal manifestations to include palatal pruritus, postnasal discharge, and persistent coughing. Based on the unified airway theory, AR is strongly associated with asthma, with 15%–38% of AR patients experiencing concurrent asthma (23, 24). Additionally, the relationship between AR and various forms of chronic rhinosinusitis has been increasingly investigated (25).While direct treatment costs for AR are substantial (8, 26), indirect costs—particularly those related to lost work productivity—far exceed direct expenditures (27, 28).

Although pharmacotherapy remains a standard treatment for AR symptom relief, its limitations, such as the need for prolonged use and potential adverse effects, diminish its long-term efficacy. Consequently, there is a growing demand for safer and more effective therapeutic alternatives. It was reported that about 27%–46% of AR patients prefered to complementary and alternative treatments for their allergic symptoms (29–31). Recent studies have demonstrated that alternative therapies can effectively relieve allergic rhinitis symptoms (32, 33),with efficacy comparable to Western medicine (34). In 2023, ICAR emphasized evidence-based therapies as primary strategies, with alternative therapies playing a potential supportive role in select cases (35).

Traditional Chinese medicine first documented AR (“Biqiu”) over 2000 years ago. The *Systematic Classic of Acupuncture and Moxibustion* (265–316 AD) records LI20 (Yingxiang) acupuncture for Biqiu treatment. Recent studies found that acupuncture may relieve both nasal and non-nasal symptoms of AR patients,improve their life quality (36–38) and in our previous parallel control studies also found that after 10 days of treatment, the scores of major nasal and eye symptoms were cumulative lower than before (13, 39). And the immediate effective rate of the first treatment of acupuncture was 52.1% (40).

The mechanism of acupuncture treatment for allergic rhinitis is still unclear, but studies suggest it may involve the following aspects: (1) Neuromodulation: Stimulation of specific acupoints may modulate both sympathetic and parasympathetic nervous system activity, potentially reducing nasal inflammation, congestion, and hypersecretion (41, 42). (2) Immunomodulation: Acupuncture may suppress inflammatory mediators (43, 44) and help restore Th1/Th2 immune balance, potentially reducing IgE-mediated allergic responses (45, 46). (3) Neuropeptide regulation: Modulation of substance *P* may reduce nasal hyperreactivity and symptom severity (43).

Now existing studies lacked control groups or combined acupuncture with other treatments, potentially confounding its efficacy in allergic rhinitis (AR). Additionally, few studies have compared acupuncture with sham acupuncture, and the lack of standardized sham methods may obscure its true therapeutic effects. To address these issues, we designed a randomized, single-blind, sham-controlled trial following <The SHARE:SHam Acupuncture REporting guidelines and a checklist in clinical trials > (16)^,^ using pragmatic placebo needles on sham acupoints to assess acupuncture's specific effects. This study will also evaluates changes in AR patients’ quality of life and may provide the time-effect relationship to optimize treatment frequency and duration. To ensure ethical and realistic efficacy assessment, participants may use anti-allergic drugs for intolerable symptoms, with detailed medication records.

This study has several limitations that to acknowledged. First, the single-center design may restrict the generalizability of our findings to other clinical settings and more diverse populations,as cultural factors, patient expectations, and practitioner expertise unique to our institution could potentially influence treatment outcomes. Additionally, while the homogeneous patient population and standardized treatment protocols at our center help ensure internal validity, they may not fully capture the variability encountered in real-world clinical practice. To address these limitations and strengthen the evidence base for acupuncture in treating perennial allergic rhinitis, future research should employ multi-center trial designs incorporating diverse healthcare settings, different cultural contexts, and practitioners with varying levels of expertise. Such studies would significantly enhance the external validity of the findings.
